# Improving pneumonia case-management in Benin: a randomized trial of a multi-faceted intervention to support health worker adherence to Integrated Management of Childhood Illness guidelines

**DOI:** 10.1186/1478-4491-7-77

**Published:** 2009-08-27

**Authors:** Dawn M Osterholt, Faustin Onikpo, Marcel Lama, Michael S Deming, Alexander K Rowe

**Affiliations:** 1 Division of Parasitic Diseases, Centers for Disease Control and Prevention, Atlanta, GA, USA; 2 Division of General and Community Pediatric Research, Cincinnati Children's Hospital, Cincinnati, OH, USA; 3 Direction Départementale de la Santé Publique de l'Ouémé et Plateau, Ministry of Health, Porto Novo, Benin; 4 Africare-Benin, Porto Novo, Benin

## Abstract

**Background:**

Pneumonia is a leading cause of death among children under five years of age. The Integrated Management of Childhood Illness strategy can improve the quality of care for pneumonia and other common illnesses in developing countries, but adherence to these guidelines could be improved. We evaluated an intervention in Benin to support health worker adherence to the guidelines after training, focusing on pneumonia case management.

**Methods:**

We conducted a randomized trial. After a health facility survey in 1999 to assess health care quality before Integrated Management of Childhood Illness training, health workers received training plus either study supports (job aids, non-financial incentives and supervision of workers and supervisors) or "usual" supports. Follow-up surveys were conducted in 2001, 2002 and 2004. Outcomes were indicators of health care quality for Integrated Management-defined pneumonia. Further analyses included a graphical pathway analysis and multivariable logistic regression modelling to identify factors influencing case-management quality.

**Results:**

We observed 301 consultations of children with non-severe pneumonia that were performed by 128 health workers in 88 public and private health facilities. Although outcomes improved in both intervention and control groups, we found no statistically significant difference between groups. However, training proceeded slowly, and low-quality care from untrained health workers diluted intervention effects. Per-protocol analyses suggested that health workers with training plus study supports performed better than those with training plus usual supports (20.4 and 19.2 percentage-point improvements for recommended treatment [p = 0.08] and "recommended or adequate" treatment [p = 0.01], respectively). Both groups tended to perform better than untrained health workers. Analyses of treatment errors revealed that incomplete assessment and difficulties processing clinical findings led to missed pneumonia diagnoses, and missed diagnoses led to inadequate treatment. Increased supervision frequency was associated with better care (odds ratio for recommended treatment = 2.1 [95% confidence interval: 1.1-3.9] per additional supervisory visit).

**Conclusion:**

Integrated Management of Childhood Illness training was useful, but insufficient, to achieve high-quality pneumonia case management. Our study supports led to additional improvements, although large gaps in performance still remained. A simple graphical pathway analysis can identify specific, common errors that health workers make in the case-management process; this information could be used to target quality improvement activities, such as supervision (ClinicalTrials.gov number NCT00510679).

## Background

Pneumonia is a leading cause of child deaths in developing countries [1,2]. While vaccination against agents such as *Streptococcus pneumoniae *and *Hemophilus influenzae *could prevent many pneumonia cases, adequate management of cases that do occur is essential to reduce pneumonia mortality. Evidence suggests that children with pneumonia often do not receive potentially life-saving antibiotics [3].

To improve the management of pneumonia and other common causes of child mortality, the World Health Organization (WHO) and other partners developed the Integrated Management of Childhood Illness (IMCI) strategy. A key component of IMCI is a set of evidence-based guidelines for classifying (diagnosing) and treating illnesses in first-level health facilities that lack sophisticated diagnostic equipment and treatments [4].

WHO recommends implementing the guidelines through an 11-day, in-service training course, a follow-up visit to health workers' facilities four to six weeks later to reinforce new practices, and job aids (e.g. a flipchart of clinical algorithms and a one-page form for recording a patient's assessments, disease classifications and treatments). For brevity, we use "IMCI training" to describe this implementation process.

More than 110 countries are implementing IMCI (personal communication, T. Lambrechts, WHO, May 21, 2007) and studies have demonstrated that the strategy can improve health care quality at health facilities [5-8] and seems to reduce mortality [9]. However, despite the favorable results, these same studies show that health workers' adherence to IMCI guidelines could still be improved, with some investigators calling attention to the need for ongoing support for health workers after IMCI training [10].

In the late 1990s, as Benin planned to introduce IMCI, concerns were raised about WHO's implementation approach. There were worries that the training would not lead to long-term changes in health worker practices and that printing an IMCI recording form for each patient would be unaffordable. To address these concerns, we designed a novel package of supports for health workers after IMCI training (see Interventions, below) and conducted a trial to measure the cost and effectiveness [11].

Because IMCI in Benin was initially implemented in the context of a disease-control project (the US Africa Integrated Malaria Initiative), which might have emphasized malaria over other conditions, and because the complexity of disease-specific portions of IMCI guidelines seemed different (e.g. management of respiratory infections seemed more complex than management of fever), we performed a series of analyses to determine whether the effectiveness of our post-training supports (and of IMCI training) varied for different diseases. Pneumonia was especially critical to study because a baseline survey in the study setting showed that care for respiratory illnesses was extremely poor-only 5.0% (7/141) of pneumonia cases were correctly classified, and no child had a complete assessment of respiratory symptoms [12].

Our objectives in this study were to: (1) evaluate the effectiveness of IMCI training and post-training supports on the quality of pneumonia case management; (2) examine specific causes of common errors in the case-management process with a simple graphical pathway analysis; and (3) identify the factors that influence case-management quality with statistical modelling.

## Methods

### Population and study design

**Figure 1:**
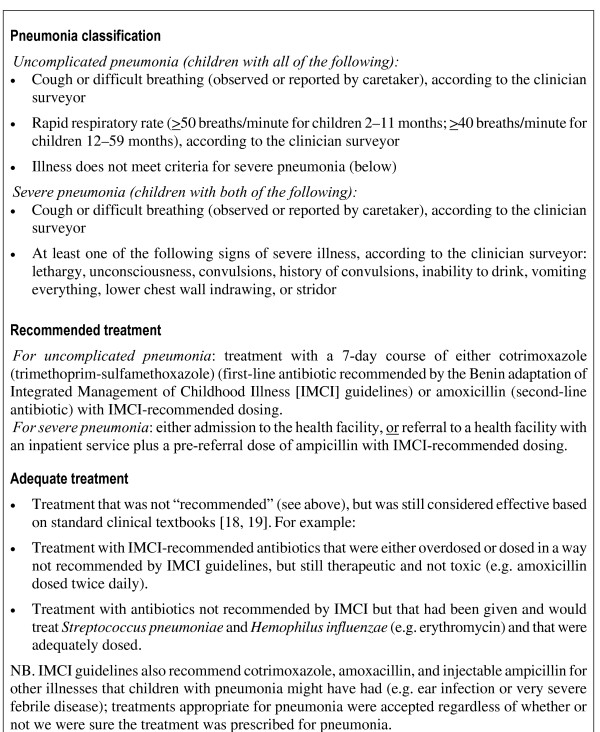
**Definitions of pneumonia classification and treatment categories**.

The study area, Ouémé and Plateau Departments (estimated 2005 population 1.2 million [13]), Benin, typifies West Africa: widespread poverty, weak infrastructure, low levels of education, endemic malaria and high child mortality [14,15]. The trial was initially designed as a before-and-after study with a randomly selected control group (see reference [11], Figure 1, for timeline). The study area was divided into two areas (i.e. two units of randomization), each comprising eight communes (see reference [11], Figure A, for map); then one area was randomly chosen as the intervention area to receive IMCI training plus study supports and the other to receive IMCI training plus "usual supports". Further details on the study design, interventions, and data collection are described elsewhere [11,12].

Due to unexpectedly slow implementation of IMCI training, many consultations were provided by non-IMCI-trained health workers. Therefore, in addition to the intention-to-treat analysis, we formulated an alternative per-protocol analysis to present as the focus of this paper. The per-protocol analysis compared consultations performed by IMCI-trained health workers with study supports (health workers who were trained and therefore received the intended intervention), IMCI-trained health workers with usual supports (health workers in the control area who were trained) and health workers who did not receive IMCI training due to the above-mentioned logistical delays.

We conducted four health facility surveys: a baseline (pre-IMCI) survey in 1999 and three follow-up surveys after IMCI implementation began (2001, 2002 and 2004). Inclusion criteria were: public and licensed private health facilities with an outpatient department in the study area, and a level of care appropriate for IMCI (i.e. one referral hospital and one subspecialty hospital were excluded). We used cluster sample surveys in which the unit of observation was an ill-child consultation; the primary sampling unit was the health facility-day (i.e. all ill children seen at a health facility during regular working hours on one weekday). Because the 2002 health facility survey was conducted only in communes where IMCI training courses had taken place, the sampling frame of this survey differed from that of the other surveys and these observations were excluded from the intention-to-treat analyses.

### Interventions

IMCI was implemented by means of WHO's approach (see Background). Although we intended to train eligible health workers in one year, due to funding and logistical problems it took four years to complete all the planned 11-day training courses (five courses were taught in 2001, two in 2002, three in 2003 and one in 2004). In 2001, only 30% of pneumonia cases were seen by IMCI-trained providers, and by 2004 the proportion had climbed to 80% (Table 1).

**Table 1:** Enrollment of study participants by year of survey

**Survey year**	**Consultations observed**	**No. of children with clinical pneumonia in analysis^a^**	**Children in analysis seen by a health worker who received Integrated Management of Childhood Illness training** **n/N (%)**
1999 (baseline)	583	114	0/114 (0)

2001 (follow-up 1)	393	82	25/82 (30.5)

2002 (follow-up 2)	231	51	21/51 (41.2)

2004 (follow-up 3)	370	54	43/54 (79.6)

Total	1577	301	

^a^Children seen for an initial consultation with a "gold standard" Integrated Management of Childhood Illness classification of pneumonia whose treatment was not undefined.

IMCI-trained health workers in the intervention area received a package of study supports: IMCI-specific supervision (we intended two contacts every three months), supervision workshops, supervision of supervisors, job aids (patient registers that replaced IMCI recording forms, and counseling guides [11]), and non-financial incentives (certificate of merit presented at a ceremony annually). All components were implemented together. Notably, however, only 29% (339/1186) of planned supervision visits actually occurred [16]. IMCI-trained health workers in the control area received "usual" supports: job aids (packets of IMCI recording forms) and some IMCI-specific supervision. Additionally, all health workers potentially benefited from five additional vehicles for supervision provided by a donor in 2002; decentralization of the health system that occurred throughout Benin (*commune *supervisors given some control over budgets); and results of our surveys, which were shared at least annually.

### Data collection

The study protocol was approved by the Ethics Committee of the Benin Ministry of Public Health and CDC's Human Subjects Review Board, and was registered with ClinicalTrials.gov (Identifier: NCT00510679). The 1999 survey was considered program evaluation and written consent was not required; verbal consent was requested from all participants (health workers and children's caretakers). Surveys from 2001-2004 were considered research, and written informed consent was requested from all participants.

After obtaining consent from health workers and child caretakers (usually the mother), we collected data with five standardized methods: (1) silent observation of consultations with a checklist; (2) caretaker interviews to ascertain prescribed medications and understanding of treatment instructions; (3) child re-examination by a study clinician to determine "gold standard" IMCI classifications; (4) health facility assessment to evaluate supplies and other attributes; and (5) health worker interviews to obtain information on demographics, training, supervision and other characteristics.

### Definitions

**Figure 2:**
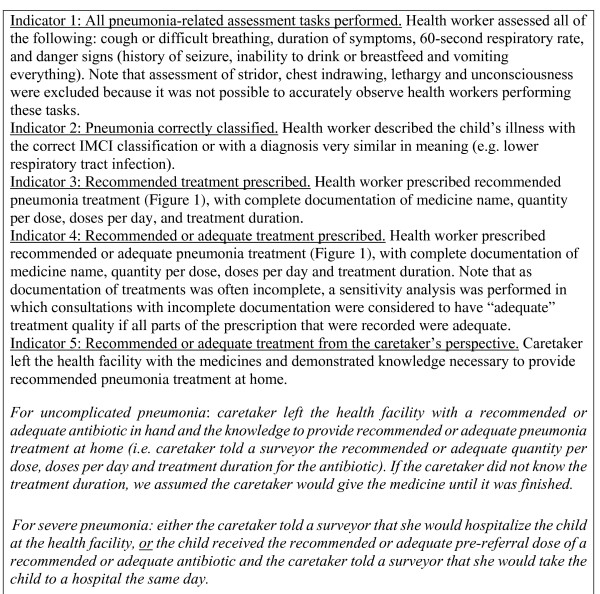
**Definitions of the indicators of pneumonia case-management quality**.

The definition of clinical pneumonia (Figure 1) was based on Benin's adaptation [17] of WHO's generic IMCI guidelines [4]. Treatments were categorized as: (1) recommended (treatment exactly matched IMCI guidelines (Figure 1)); (2) adequate (treatment not recommended, but still considered effective based on standard clinical textbooks) [18,19]; (3) inadequate (neither recommended nor adequate); or (4) undefined (children with uncomplicated pneumonia who needed urgent referral for another problem, as IMCI recommends that treatment of non-severe illnesses such as uncomplicated pneumonia should not delay urgent referral for severe illnesses). Conceptually, recommended, adequate and inadequate treatment correspond to "no error," "minor error" and "major error," respectively [20]. Outcome indicators are defined in Figure 2. Outcome indicators for a sensitivity analysis were created that accounted for incomplete documentation of health worker prescriptions (Figure 2, indicator 4).

### Analysis

Data were double-entered and verified using EpiInfo software [21]. Analyses were restricted to ill children 2-59 months old seen for an initial consultation with a "gold standard" IMCI classification of pneumonia (uncomplicated or severe) and a treatment that was not undefined (see Definitions). Analyses were performed with SAS version 9.1 software [22]. Hypothesis testing and confidence interval (CI) estimation were done with an alpha level of 0.05.

For each outcome, a logistic regression model was constructed that contained indicator variables for time (early or late follow-up period versus baseline), study area (IMCI intervention or control), and area-time interactions. The interactions, which compared time trends between intervention and control areas, were the main effects. Models were constructed with the SAS GENMOD procedure, which uses generalized estimating equations, with an exchangeable working correlation matrix to account for correlation in the data.

Given that IMCI training happened slowly and that quality measures in both study areas were likely diluted by consultations provided by non-IMCI trained health workers, we felt that the results of the intention-to-treat analysis did not capture the full results of the trial. To further evaluate the effectiveness of IMCI training and the post-training supports (Objective 1), three health worker groups were compared: IMCI-trained residing in the intervention areas where study supports were provided; IMCI-trained residing in control areas where usual supports were provided; and non-IMCI-trained residing in either study area.

The number of pneumonia cases in each of the follow-up surveys was relatively small, therefore all three follow-up surveys were combined. Models were constructed similar to those used in the intention-to-treat analysis, except the indicator variable that coded for study group was replaced by two indicator variables that coded for the three health worker groups (IMCI with study supports, IMCI with usual supports and no IMCI). The health worker group-time interactions, which compared time trends between health worker groups, were the main effects.

We evaluated 17 factors (e.g. caseload, demographic factors and clinical features) as potential confounders of the health worker group-outcome association by entering factors into models one at a time. Factors thought to be in the causal pathway between the intervention and correct treatment (e.g. correct diagnosis) were not considered. Factors that changed model estimates by >20% without causing model instability were considered confounders and retained in the final model [23]. Effect sizes defined as absolute percentage-point (%-point) "difference of differences" (e.g. [follow-up - baseline]_IMCI/studysupports _- [follow-up - baseline]_IMCI/usualsupports_) were estimated with predicted probabilities from the logistic regression models at baseline and follow-up time points for each of the health worker groups, with confounders held constant.

The above effect sizes require an estimate of baseline (pre-IMCI) outcome values for each of the health worker groups. These values were estimated by dividing the 16 *communes *in the 1999 survey into three parts: four IMCI pilot *communes *in the intervention area (baseline for the IMCI/study supports group), four IMCI pilot *communes *in the control area (baseline for the IMCI/usual supports group), and eight non-IMCI-pilot *communes *(baseline for the no-IMCI group). For details, see Figure 1 and Figure A of reference [11].

To examine specific causes of common errors in the case-management process (Objective 2), we used a simple graphical pathway analysis. In quality improvement methodologies, this is conceptually similar to a "root-cause" analysis [24]. We began with the ideal case-management pathway. IMCI guidelines require health workers to: (1) assess the child; (2) classify respiratory illnesses as "no pneumonia: cough or cold", uncomplicated pneumonia or severe pneumonia; and (3) treat the child (for uncomplicated pneumonia cases, treat with antibiotics, appropriately dosed and documented). For the 70 children with uncomplicated pneumonia and defined treatment quality, we constructed a flow diagram that summarized the case-management pathways that actually occurred and thus showed how health workers deviated from ideal (complete assessment → correct diagnosis → correct treatment). To focus on the most serious errors (no antibiotic or under-dosed antibiotic), recommended and adequate treatment were combined.

To identify the factors that influenced case-management quality (Objective 3), we studied the 70 children with uncomplicated pneumonia seen by IMCI-trained health workers whose treatment quality was defined. We assessed three health facility factors, 26 health worker factors and 21 child/consultation factors for their association with recommended treatment and "recommended or adequate" treatment. A forward-stepwise modelling approach was used to construct multivariate logistic regression models [23,25]; correlation was accounted for with methods described above.

## Results

### Enrolment

Altogether 1577 ill-child consultations were observed in the four health facility surveys (Table 1), including 1244 initial consultations. Initial consultations were observed during 301 visits (each lasting one day) to 114 different health facilities (some visited more than once) and performed by 267 health workers (for details, see Table 2 of reference [11]). Of 366 initial consultations in which the child had clinical pneumonia, 301 were included in the per-protocol analysis; 65 were excluded because treatment was undefined (see Definitions). These 301 consultations took place in 88 health facilities (68 small public facilities, 13 large public facilities or outpatient departments of district hospitals, and seven private or religious health facilities). Consultations were performed by 128 health workers (22 nurse's aides, 97 nurses and nine physicians). The 51 consultations from the 2002 health facility survey were excluded from the intention-to-treat analysis because of the previously mentioned differences in sampling strategy. Further details on enrolment and study group characteristics are presented elsewhere [11].

**Table 2:** Predictors of pneumonia^a ^treatment practices of health workers trained in IMCI

		**Recommended or adequate treatment**		
		
**Characteristic**	**No. of consultations or mean value**	**OR (95% CI)**	**n (%)**	**OR (95% CI)**
*Final multivariate models*				

Health worker received				
**Study supports**	N = 28	**3.0 (1.0, 8.6)**	15 (53.6)	1.5 (0.6, 3.7)
Usual supports	N = 42	ref.	19 (45.2)	ref.

No. supervisory visits, past 6 months (ranging from 0-4)	mean = 0.9	**2.1 (1.1, 3.9)**	--	**1.6 (1.1, 2.3)**

Consultation duration, in minutes (ranging from 5 to 131)	median = 16	**1.04 (1.00, 1.08)**	--	--^b^

No. of IMCI classifications (ranging from 1 to 4)	mean = 2.8	--^b^	--	**2.0 (1.2, 3.3)**

*Causal pathway variable omitted* *from multivariate modelling*				

Health worker correctly diagnosed pneumonia				
Yes	N = 49	**49.8 (5.6, 442.1)**	28 (68.3)	**14.2 (4.0, 50.3)**
No	N = 21	**ref**.	6 (20.7)	**ref**.

^a ^Seventy children seen for an initial consultation with a "gold standard" IMCI classification of uncomplicated pneumonia whose treatment was not undefined (see Methods).

^b ^Variable not retained in the multivariate model.

CI = confidence interval, OR = odds ratio, ref. = reference level

### Effect of study supports and IMCI training

**Figure 3:**
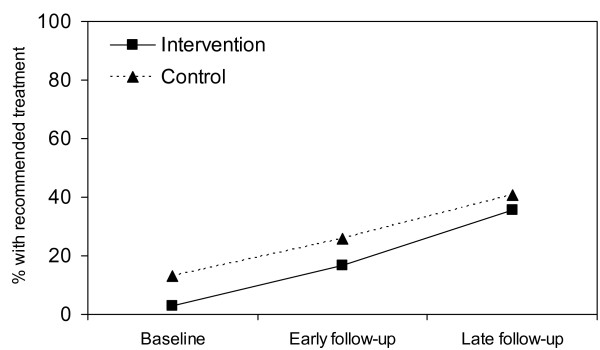
**Intention-to-treat analysis of the effect of post-training supports on recommended treatment**.

**Figure 4:**
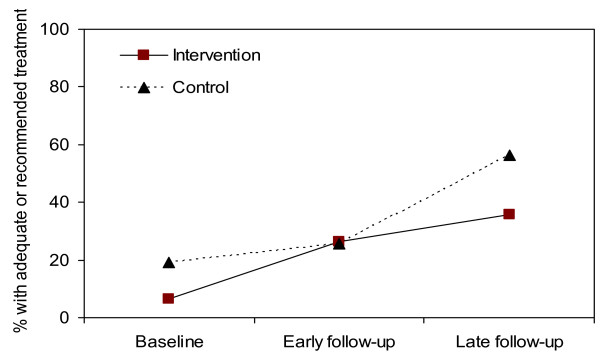
**Intention-to-treat analysis of the effect of post-training supports on adequate or recommended treatment**. IMCI = Integrated Management of Childhood Illness. P-value early follow-up v. baseline = 0.27. P-value late follow-up v. baseline = 0.17. P-value early follow-up v. baseline = 0.16. P-value late follow-up v. baseline = 0.66. Models are adjusted for correlation, however no confounding.

In an analysis based on the original randomized-controlled study design (i.e. intention-to-treat analysis), treatment quality improved over time for both primary outcomes, although differences in improvements between the study supports area and usual supports area were not statistically significant (Figures 3 and 4). However, as previously mentioned, IMCI training proceeded slowly; and low-quality care from non-IMCI-trained health workers diluted intervention effects (see Table 1).

Results of the per-protocol analysis are presented in Additional file 1. Effect sizes and p-values in columns 8-9 compare case-management quality of the IMCI/study support group versus the IMCI/usual support group-i.e. the effect of study supports. Effects and p-values in columns 10-11 compare quality of the IMCI/usual support group versus the no-IMCI group-i.e. the effect of IMCI training. The five indicators in Additional file 1 represent different aspects of the case-management process: assessment of the patient, diagnosis, treatment and counselling. Our main outcomes of interest were indicators 3 (recommended treatment prescribed) and 4 (recommended or adequate treatment prescribed). Study groups were similar on most characteristics (e.g. health facility type, medicine availability, health worker pre-service training, child's age and illness severity); and based on our analysis to identify confounding, the few differences that were seen were unlikely to bias effect sizes (data not shown).

**Figure 5:**
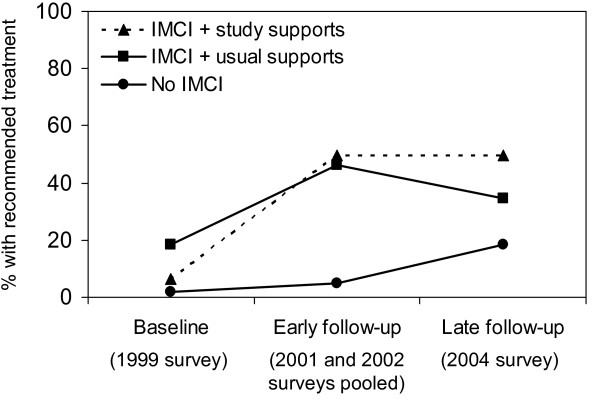
**Per-protocol analysis: effect of IMCI training plus study supports and IMCI training plus usual supports on recommended treatment predicted probabilities from adjusted model^a^**.

For recommended treatment, improvements in the IMCI/study supports group were 20.4%-points greater than the IMCI/usual supports group, although this result was of borderline statistical significance (p = 0.08) (Additional file 1, row 3, columns 8-9). That is, the results of the per-protocol analysis suggest that the study supports were associated with greater improvements in treatment quality. A comparison of the IMCI/usual supports group with the no-IMCI group showed no significant effect of IMCI training (effect = 18.1%-points, p = 0.90). When the follow-up period was divided into early follow-up (2001-2002 surveys combined) and late follow-up (2004 survey), no statistically significant effect was found for either study supports or IMCI training (Figure 5). Though the figure appears to show a secular trend toward better care among untrained health workers, this trend was not statistically significant.

**Figure 6:**
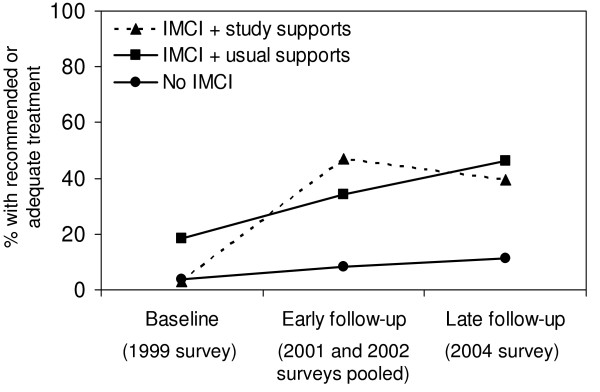
**Per-protocol analysis: effect of IMCI training plus study supports and IMCI training plus usual supports on "recommended or adequate" treatment, predicted probabilities from adjusted model^b^**. IMCI = Integrated Management of Childhood Illness. ^a^Model adjusted for correlation (no confounders). P-values comparing the IMCI/study supports group with the IMCI/usual supports group were 0.15 (early follow-up versus baseline) and 0.10 (late follow-up versus baseline). P-values comparing the IMCI/usual supports group with the no-IMCI group were 0.73 (early follow-up versus baseline) and 0.29 (late follow-up versus baseline). ^b^Model adjusted for correlation, availability of inpatient service, and severe pneumonia (the two confounders were held constant with the values *no inpatient service *and *non-severe pneumonia*). P-values comparing the IMCI/study supports group with the IMCI/usual supports group were 0.01 (early follow-up versus baseline) and 0.08 (late follow-up versus baseline). P-values comparing the IMCI/usual supports group with the no-IMCI group were 0.96 (early follow-up versus baseline) and 0.87 (late follow-up versus baseline).

For "recommended or adequate" treatment (Additional file 1, row 4), improvements in the IMCI/study supports group were 19.2%-points greater than the IMCI/usual supports group (p = 0.01). That is, the study supports were associated with improved treatment quality. No significant effect was found for IMCI training (effect = 16.7%-points, p = 0.79). Results were significant or borderline significant when the follow-up period was divided into early and late follow-up (Figure 6).

### Treatment quality by IMCI-trained health workers

In follow-up surveys, among 89 children with pneumonia and defined treatment quality seen by IMCI-trained health workers, 43.8% received recommended treatment (63.2% [12/19] for severe pneumonia; 38.6% [27/70] for uncomplicated pneumonia); 9.0% received adequate but not recommended treatment (5.3% [1/19] severe; 10.0% [7/70] uncomplicated); and 47.2% received inadequate treatment (31.6% [6/19] severe; 51.7% [36/70] uncomplicated). The next two sections present in-depth analyses that explore reasons for correct treatment and errors in the management of the 70 children with uncomplicated pneumonia and defined treatment quality.

### Graphical pathway analysis for IMCI-trained health workers

**Figure 7:**
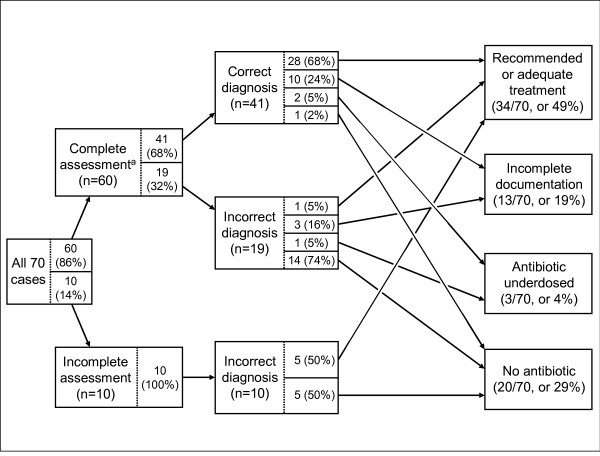
**Pathway analysis in 70 cases of non-severe pneumonia treated by IMCI-trained health workers**. ^a^Complete assessment means health worker ascertained that the child had cough or difficult breathing (i.e. health worker asked for the symptom or the caretaker spontaneously offered it) and counted the child's respiratory rate.

This analysis (Figure 7) revealed four primary findings. First, incorrect diagnosis was a key problem, as it preceded two thirds (23/36) of all treatment errors; nearly all (19/20) children who received no antibiotics were incorrectly diagnosed. Once correctly diagnosed, failure to prescribe an antibiotic was unusual. Second, incomplete documentation was a problem, accounting for one third (13/36) of all errors. As incomplete documentation could leave pharmacists and caretakers less sure of how to give a medication, inadequate treatment might result. Third, although numbers are small, it is notable that half (5/10) of the children with incomplete assessment and incorrect diagnosis still received recommended or adequate treatment, usually without an identifiable indication. Finally, under-dosing of antibiotics was rare, accounting for only 8% (3/36) of all errors.

### Predictors of correct pneumonia treatment among IMCI-trained health workers

The 70 children with uncomplicated pneumonia and defined treatment quality were seen by 44 IMCI-trained health workers (19 health workers with study supports, 24 with usual supports and one who spent time in areas with and without study supports). To screen hypotheses in an exploratory analysis of which factors influence correct treatment for pneumonia, we used logistic regression modelling to examine 44 factors for their association with treatment quality.

Unfortunately, several factors of particular interest could not be studied because of a lack of variability: pre-service training (nearly all health workers were nurses), health facility type (there were comparatively few private health facilities), job aids (most health workers used them) and health worker knowledge (mean score of a knowledge assessment based on case scenarios was 97%). By exclusion, these factors were unlikely to confound the associations reported below.

For recommended treatment (Table 2, columns 3-4), the multivariate model revealed that children seen by health workers who received study supports had threefold greater odds of receiving recommended treatment (p = 0.047); each supervisory visit doubled the odds (p = 0.025) and each extra minute of consultation duration increased the odds by 4.2% (p = 0.028). Correct diagnosis, which was excluded from the multivariate analysis because it was considered a causal pathway variable, was strongly associated with recommended treatment (Table 2, last row).

For recommended or adequate treatment (Table 2, columns 5-6), the multivariate model revealed that the only statistically significant associations were with increasing number of supervisory visits and increasing number of IMCI classifications. These associations were not present in the sensitivity analysis that accounted for incomplete documentation of prescriptions. As with recommended treatment, correct diagnosis was strongly associated with recommended or adequate treatment. Study supports were not associated with the outcome.

Among the many factors not statistically significantly associated with treatment quality, several were of particular interest: drug availability, IMCI-trained colleague in the health facility, time since IMCI training, years of experience, primary language of caretaker and health worker being different, child's respiratory rate and chief complaint of cough or difficult breathing.

## Discussion

The quality of pneumonia case management in Benin before IMCI was extremely poor; over the four-year study, quality improved. The comparison of the IMCI/usual supports group with the no-IMCI group showed that IMCI training was associated with better assessment and pneumonia classification, but not with better treatment (the IMCI/usual supports group gave correct treatment more often, but the result was not statistically significant). We also demonstrated a statistically significant 19.2%-point effect of the study supports for adequate or recommended treatment, and a similar but borderline-significant (p = 0.08) trend for recommended treatment. These results suggest that to improve treatment quality, a one-time training input has less impact than training coupled with continued support, as in our study.

We found diverse results for improvements in case-management quality for different important conditions in Benin. Improvements were seen with IMCI training for all outcomes studied (pneumonia treatment, malaria treatment, anaemia treatment and a summary of case management for all conditions) [11]. However, improvements for pneumonia treatment were lower than for the other outcomes, specifically for malaria treatment (unpublished data). This raises the possibility that the context of IMCI implementation in our study (i.e. a malaria control project) might have affected the quality for non-malaria illnesses-for example, by inadvertently de-emphasizing pneumonia case management. Perhaps even more likely, IMCI's pneumonia sub-algorithm was more difficult than other parts of IMCI guidelines. Given this complexity, we thought it important to explore pneumonia treatment errors in-depth.

### Case-management quality among IMCI-trained health workers

The in-depth examination of errors by IMCI-trained health workers via graphical pathway analysis allowed us to pinpoint problems in how health workers applied the guidelines, and thus gives a view into the decision-making process we have not previously seen in the published literature. In 40% of the 70 non-severe pneumonia cases, all aspects of care (assessment, classification, and treatment) were adequate. In the remaining 60% of cases with problems, we found that errors were not uniformly distributed throughout the algorithm, but were grouped in several specific points; identifying these error points led to specific recommendations for improvement.

For example, not surprisingly, missing the pneumonia diagnosis preceded virtually all major errors (no antibiotic prescribed). Of the 29 missed diagnoses, one third could be attributed to incomplete assessment (which always led to a missed diagnosis), and two thirds could be attributed to health workers' misinterpreting clinical signs and symptoms or incorrectly processing clinical data into a diagnosis. Another example was that incomplete documentation, which could confuse pharmacists and caretakers, was relatively common. The analysis also revealed that some potentially important problems, such as under-dosing antibiotics, were rare. These results could direct supervision and other efforts to focus on complete assessments, correctly processing clinical data into diagnoses and full documentation of prescribed medicines.

Multivariate modelling showed that study supports, supervision visits, longer consultation duration and a greater number of IMCI classifications were associated with at least one measure of treatment quality, although only supervision was associated with both outcomes.

Supervision, a key component of our intervention, was associated with health care quality in a dose-response relationship. This finding agrees with other studies [25] and supports its continued use in our setting. Our baseline survey, however, found that supervision was not associated with improved pneumonia treatment [12]. While the quality of that earlier supervision was unknown, the present analysis is among health workers who received at least some supervision from staff trained by our team specifically to provide supportive supervision. Thus, our results illustrate that high-quality supervision is associated with better care.

Longer consultation duration was associated with better adherence to IMCI guidelines, but the direction of causality is unclear. Better-performing health workers could be taking more time with patients. Alternatively, given ample time to spend with patients, health workers might perform better. Though not significant in multivariate modelling, univariate results showed that lower caseloads were associated with better health care quality, possibly supporting the latter explanation. A recent time-motion study of IMCI-trained physicians in Brazil found that caseload was inversely associated with consultation time, with the association being strongest at caseloads over 50 per day, and that quality of care was highest in the areas where health workers spent, on average, more time with each patient [26]. Regardless of the direction of causality, it is clear that high-quality care requires sufficient time for each patient.

Our multivariate analyses revealed that an increasing number of IMCI classifications (diagnoses) were associated with better pneumonia treatment quality. This finding differs somewhat from other analyses in this cohort (unpublished data). Taking all consultations together-not just pneumonia cases-we found that children with more IMCI classifications and more-complex cases generally received poorer quality care, in a linear fashion.

One explanation for the different finding among the subgroup with pneumonia might be that antibiotics for pneumonia have many uses and might be more often overused than other IMCI medications (e.g. antimalarials, oral rehydration solution, iron and vitamin A). Figure 7 shows that even in consultations where children were not diagnosed with pneumonia, some health workers gave antibiotics. Therefore the association between more classifications and better treatment might reflect "the right treatment for the wrong reason" rather than the greater number of classifications somehow directly causing health workers to adhere to guidelines more carefully.

Finally, knowledge of pneumonia case management was very high among IMCI-trained health workers, despite fairly poor care being delivered. This finding is a striking example of the knowledge-practice gap that has been observed in other settings [27] and might help explain why IMCI training alone was not associated with better treatment.

### Limitations and methodological challenges

First, the sample of pneumonia cases was relatively small, and the intervention was not fully implemented for all health workers. Second, with health workers being trained over several years, our cross-sectional surveys did not allow us to evaluate a single cohort of health workers over time. However, a re-analysis of these data by time since training still provided fairly robust evidence that performance did not deteriorate up to three years after training.

Third, the study was initially planned as a group-randomized trial, and due to implementation problems, the data presented are from per-protocol analyses that stratified subjects by intervention exposure-an analytic approach recommended by some experts [28]. Fourth, pneumonia was not the main focus of the project, nor of data collection; therefore the importance of pneumonia within IMCI courses might have been inadvertently de-emphasized; with the small number of pneumonia cases, power to detect associations may not have been present, and some case characteristics useful for studying pneumonia case management might not have been collected.

Fifth, our use of group randomization with only two groups was unlikely to have prevented bias from unknown factors and did not result in groups with equal baseline quality of care. Moreover, the robustness of the statistical results might have been affected. Sixth, the observation of consultations could have influenced health worker practices, perhaps overestimating quality somewhat [29], although this influence would likely have affected all study groups similarly and thus would probably not have biased effect sizes much.

Finally, incomplete documentation of prescriptions was a considerable problem. A sensitivity analysis, which assumed adequate treatment quality for missing information, showed some differences from the main analysis (a larger effect of study supports of borderline statistical significance, and a negative effect of IMCI training of borderline statistical significance [results not shown]). This issue raises an important question for researchers studying quality of care-especially for those doing direct observation studies. Should we ask health workers about missing prescription information and potentially introduce bias toward better quality, or should we remain silent observers and potentially accept uncertainty in our measures of quality?

## Conclusion

Our results add to a growing body of literature indicating that carefully designed interventions can improve health worker performance in low-resource settings, but that considerable attention must be paid to supporting health workers beyond one-time investments in training. The difficulties we encountered with training and supervision underscore the challenges of scaling up even the most basic components of a quality improvement intervention.

Though quality of care for the condition studied here remained relatively low, with no group treating more than 56% of children correctly, care did improve over time, and gains in quality were sustained. Considerable attention in future research must be paid to attributes of interventions that are scaleable and that lead to quality improvement within the context of programmes in real-world settings. Additionally, we have shown how a simple method (graphical pathway analysis) can identify specific, common errors that health workers make in the case-management process; this information could be used to target quality improvement activities, such as supervision.

## Competing interests

The authors declare that they have no competing interests.

## Authors' contributions

FO, ML, MD and AR conceived of and planned the study and interventions; FO, ML and AR conducted surveys and collected data; DO and AR performed analyses and wrote the initial draft of the manuscript; all authors worked on interpreting the results and finalizing the manuscript.

## Supplementary Material

Additional file 1 **Per-protocol analysis: effect of study supports and IMCI training on the quality of pneumonia management**. Table presents results for effectiveness of study supports and IMCI training on quality of care according to 5 different measures of quality of pneumonia case management. Effects are a percentage point improvement in the proportion of cases with correct management. MS Word table in landscape orientation. Click here for file
