# Supplementary material for: Improving pneumonia case-management in Benin: a randomized trial of a multi-faceted intervention to support health worker adherence to Integrated Management of Childhood Illness guidelines
Source: Hum Resour Health. 2009 Aug 27;7:77. doi: 10.1186/1478-4491-7-77 (PMC2752268; doi:10.1186/1478-4491-7-77)
Supplement: Additional file 1 — Per-protocol analysis: effect of study supports and IMCI training on the quality of pneumonia management. Table presents results for effectiveness of study supports and IMCI training on quality of care according to 5 different measures of quality of pneumonia case management. Effects are a percentage point improvement in the proportion of cases with correct management. MS Word table in landscape orientation. [file 1478-4491-7-77-S1.doc]

| **Indicator of pneumonia case**  **management quality** | **% of children in each treatment category** | | | | | | | |  | **Effect size (percentage-point differences)** | | | | |
| --- | --- | --- | --- | --- | --- | --- | --- | --- | --- | --- | --- | --- | --- | --- |
|  | **IMCI training + study supports** | |  | **IMCI training + usual supports** | |  | **No IMCI training** | |  | **Effect of study supports (IMCI training + study supports vs. IMCI training + usual supports)** | |  | **Effect of IMCI training (IMCI training + usual supports vs. no IMCI)** | |
|  | Baseline | Follow-up |  | Baseline | Follow-up |  | Baseline | Follow-up |  | Effect^a^ | P-value |  | Effect^a^ | P-value |
| *No. of pneumonia cases^b^* | *N=31* | *N=34* |  | *N=33* | *N=55* |  | *N=50* | *N=98* |  |  |  |  |  |  |
| Indicator 1. All pneumonia-related assessment tasks performed^c,d^ | 0 | 61.8 |  | 0 | 38.2 |  | 0 | 0 |  | 23.6 | 0.12 |  | **38.2** | **<0.0001** |
| Indicator 2. Pneumonia correctly classified^c^ | 6.5 | 67.7 |  | 6.1 | 47.3 |  | 8.0 | 12.2 |  | 23.6 | 0.30 |  | **36.0** | **0.046** |
| Indicator 3. Recommended^c^ pneumonia treatment prescribed | 6.5 | 50.0 |  | 18.2 | 40.0 |  | 2.0 | 6.1 |  | **20.4** | **0.08** |  | 18.1 | 0.90^e^ |
| Indicator 4. Recommended or adequate^c^ pneumonia treatment prescribed | 6.5 | 52.9 |  | 27.3 | 52.7 |  | 6.0 | 14.3 |  | **19.2** | **0.01** |  | 16.7 | 0.79^e^ |
| Indicator 5. Caretaker’s report of instructions was recommended or adequate treatment^c^ | 19.4 | 44.1 |  | 45.5 | 50.9 |  | 16.0 | 30.6 |  | 19.5 | 0.11 |  | –10.3 | 0.26 |

[Please remove the extra columns.]

^a^ “Difference of differences” effect sizes based on predicted probabilities from per protocol models (see Methods). E.g. for indicator 4, column 8, the value 19.2 %-points equals improvement in treatment quality in the IMCI/study-supports group from baseline to follow-up (raw values: 52.9% – 6.5%, or 46.4 %-points) minus improvement in the IMCI/usual supports group from baseline to follow-up (raw values: 52.7% – 27.3%, or 25.4 %-points). Note that predicted probabilities are slightly different from raw indicator values in columns 2–7 (e.g., the 19.2 %-point effect size does not exactly equal 46.4 %-points – 25.4 %-points). The model’s “time x study supports” interaction term was statistically significant (from column 9, the p-value = 0.01). For indicators 2 and 4, the model was adjusted for availability of inpatient service, and severe pneumonia (assuming no inpatient service and non-severe pneumonia); for all other indicators, models had no confounders. Bold type indicates results with a P-value <0.10.

^b^ Children seen for an initial consultation with a “gold standard” IMCI classification of pneumonia whose treatment was not undefined (see Methods).

^c^ See Boxes 1 and 2 for detailed definitions.

^d^ Multivariable modeling could not be performed because indicator values were zero; effect sizes were estimated from raw values of indicators (columns 2–7). The P-value in column 9 was from a GENMOD logistic regression model that adjusted for correlation and only included a term for study supports versus usual supports; the model was run on a dataset that excluded the no-IMCI group and all baseline values, as indicator values were all zero. The P-value in column 11 was from Fisher’s exact test (i.e. correlation ignored) that excluded baseline values, as indicator values were all zero.

^e^ Although the effect sizes of IMCI training and the study supports are similar, the p-value of the effect size of IMCI training is much greater than for the study supports. The very low baseline value for the no-IMCI group, coupled with a secular increase that is moderately large in relative terms (but small in absolute terms), causes the secular increase in the IMCI/usual-supports group to be similar to the secular increase in the no-IMCI group; and thus the p-value of the interaction term, which compares these two secular trends, approaches one. Although the high p-values mean that the results are not statistically significant, the effect size for IMCI training might not be simply a result of random variation.
